# Etiology of Dental Caries

**Published:** 1884-03

**Authors:** 


ARTICLE III.

### ETIOLOGY OF DENTAL CARIES.

Dr. Smith: Since our last meeting I know of nothing especially new that has been developed relating to this subject. In June last Prof. Watt delivered a very interesting address before the Indiania State Dental Society upon the causes of dental caries, in which he set forth the same views embodied in the paper which he read before us last December.

In January Dr. Miller contributed an able paper to the *Dental Cosmos*, entitled "Agency of Micro-Organisms in Decay of Human Teeth." I am pleased to say that Dr. Miller is a native of our State, and as an Ohio man I think we have reason to feel proud of him. As an earnest and original investigator regarding the causes of dental caries he to-day occupies the foremost place.

I will, if you please, call attention to some of the differences, as I understand them, in the views as recently announced by Dr. Miller, and those usually taught and accepted by the profession in our State. Dr. Miller believes that the first stage of dental caries is caused by acids, and that by far the greater part of this decalcification is caused by acids produced in the mouth by fermentation, as lactic, acetic, butyric, &c. It will be noted that he leaves out in the process the mineral acids—nitric, hydrochloric and sulphuric. Magitito, as you will remember, also omitted these acids in making the laboratory experiments, the results of which he gives in his able work on dental caries; for the reason, he says, they are rarely found in the mouth. In fact, most writers on dental pathology, as far as I know, only refer to the action of the mineral acids upon the teeth, as the effects of these agents when administered as medicines. If the three mineral acids are excluded as active agents of dental caries, of course, the three distinct varieties usually attributed to them, if present, must result from other and accidental causes.

Dr. Miller calls attention to the fact that meat in the fermative stage does not give an acid reaction as commonly supposed, but an alkaline or neutral reaction. It is only the starchy foods, then, that give the decided acidity that we observe in cavities in carious teeth. It should be noted, also, that Dr. Miller states he is not aware of a single alkaline or neutral substance which has the power in any degree to extract the lime salts from tooth structure.

In regard to the presence of micro-organisms in carious dentine, Dr. Miller is quite emphatic in stating that they are always present. The examination of over one thousand specimens of dentine affected by caries showed fungi present in every instance. Whether or not bacteria have the ability to penetrate beyond the layer of tissue softened by acids appears yet to be an open question. When these organisms are observed beyond the softened layer, their presence is explained by the supposed imperfections in the dentine, interglobular spaces, etc. Ten years ago Lebar and Rottenshue contended that the leptothrix is an important factor in the process of dental caries, but were unable to decide if these organisms could penetrate sound dentine. The attention of investigators is now being directed to this phrase of the question, and it is to be hoped that soon we shall have more definite knowledge as to the invasive habits of the fungi found in carious teeth. And in the study and investigation of this to us all-important subject of the etiology of dental caries, I would welcome any one who gave promise of helping—whether of our own guild or not. The orator in his eulogy of Lebig recently, mentioned the important service which the great chemist rendered mankind in his investigations in the department of agriculture, whereby the occupation was raised from the crude methods of the earlier time to that of an art based on scientific principles. And yet Lebig never held a plow or owned and acre. Pasteur, up to the time he began to investigate the parasitic diseases of the silk worm, had never seen such a worm. When he made his first communication on the subject to the Academy of Science, a cloud of criticism was raised. The physician and biologist said, here is a chemist rashly quitting his own sphere and presuming to lay down the law for us. Pasteur was indifferent and patiently continued his work with the result that the silk industry in France was saved from annihilation. No, dental science is not the exact science yet, that we can afford to discourage careful investigations bearing upon it, come from what source they may. I have referred to these instances of experimental work by Lebig and Pasteur as examples of what sometimes may be accomplished by outside assistance.

I wish, Mr President, to speak for a moment of chemical erosion of the teeth, not so much to discuss its etiology as to inquire of members their methods of treatment. It is generally agreed that this so-called variety of dental caries is caused by lactic or butyric fermentation of starchy and saccharine substances which find ready lodgement about the neck and other surfaces of teeth, where lesions of this kind occur. Acids being the active agents in causation, ant-acids in treatment would be naturally indicated. Prepared chalk I have frequently used to change the reaction. If used persistently, the hyper-sensitiveness of the dentine is allayed, and progress of the disease somewhat retarded; but unless we get a reactionary effect through the tissues of the teeth, no permanent benefits come from ant-acid treatment. I have had better success in the use of nitrate of silver. The somewhat persistent film formed by the union of the caustic with the organic part of the tooth, affords a protection after the manner of a filling placed over the affected territory. Very recently, upon the theory that sulphuric acid carbonizes the organic portion of the tooth and forms an insoluble compound with the calcium sulphate, as shown, it is claimed, in the mechanism of the production of caries of the black variety I applied acid to a tooth attacked by erosion. I was disappointed in not getting the black coloration promptly. I am satified now that I did not use the acid sufficiently concentrated. Only a highly concentrated acid, chemists state, will convert organic tissue into charcoal. It is questioned, you know, If we ever find sulphuric acid of the required strength, and for a sufficient period in contact with tooth structure in the mouth, to cause charing of the organic portion. If used in treatment of erosion, of course, the strength of the application is under our control. I shall watch the case I have referred to and report results obtained by the use of sulphuric acid. But, after all, are we not too apt to regard this disease as local and to be cured by topical remedies. Frequently it appears to be systemic, and then the wasting of the teeth can only be arrested by a change in the general tonicity of the system. It is well occasionally, I find, to send patients to the seaside for a cure. The question occurs to the practitioner in observing some cases, if decalcification of the affected tooth does not take place, induced by the inflammatory or irritant effects of the agent acting on the surfaces. If we may have a reactionary effect in dentine, induced through the pulp to resist the encroachment of disease, it will appear only reasonable that when nutrition is below normal we should have resorption of the hard portion of the tooth. The etiology and pathology of this superficial wasting of the teeth is not, then, quite so well understood as I intimated in the beginning of my remarks. For myself, I am free to acknowledge my inability, frequently, to control the disease to a degree that is satisfactory.

The late investigations relating to the agency of fungi in dental caries have done good in directing our attention to the comparative value of disinfectants and as remedies in the practice of dentistry. Creosote and carbolic acid still hold a prominent place in our materia medica, preference being given of late to the latter, because of its supposed superiority as a disinfectant. But it is quite probable that the chief value of carbolic acid combined with glycerine. It has been shown that this mixture is practically inert when used as an antiseptic.

The cause of the recurrence of decay in the layer of softened dentine in deep seated caries, supposed to have render asceptic before filling, as mentioned by Dr. Butler, are not quite apparent. This occurs sometimes under the best plastic fillings where leakages are precluded. We have a return here of the putrefactive state, either from the failure of the persistent effects of the antiseptic, or else the germ is brought in contact with the tissue by the vascular circulation in the pulp. At our last session I referred to cases in which the contents of pulp cavities undergo putrefactive decomposition, though the pulps had never been exposed to the air. If the doctrine, no potential life, no putrefaction is accepted as final, these examples appear to be phenomenal.

The study of the processes of fermentation and putrefaction impresses us with the need of the utmost care in treatment and manipulation, if we wish to produce asceptic results. To treat issue with antiseptics and then let the fluids of the mouth come in contact with the part before isolation, as frequently done, is certainly not observing proper "antiseptic precaution." And if we believe Tyndall in the relations of dust and disease, there are other precautions to be observed besides the exclusion of saliva from the cavity of decay.

Dr. Berry: Dr. Watt teaches that when nitrogenous substances putrify, ammonia results, and that it causes precipitation of the lime salts and white decay of the teeth. I do not think the theory that bacteria act on the teeth causing them to decay is worthy of much consideration.

Dr. Taft: Etiology means a discussion or treatise upon the causes of things. The question here is, what are the causes of decay of the teeth? I suppose all know they are divisible into two classes, those that predispose teeth to decay, and those that produce the decay. Let us bear in mind that these two divisions are clear and distinct. There are many things which predispose teeth to decay, but perhaps we know more of exciting causes than of predisposing causes. Where the structure is imperfect there are more likely to decay than where they are perfect. Again, the teeth may be comparatively well organized but, owing to defective nutrition, they may be likely to decay. Then, enfeeblement of the vital forces predisposes the teeth to decay. And that leads us to the proposition that teeth, the pulps of which have been destroyed, are more liable to decomposition than live teeth. In the teeth of very young persons this breaking down will occur much more readily than later in life. There are often openings through the enamel, through which decay will be likely to occur of more or less marked character, sometimes just perceptible under the microscope, at other times so marked as to be seen by the naked eye. In regard to the exciting causes there has been much discussion, and various opinions are entertained on the subject. Some things about decay of the teeth are obscure. But we understand that simple chemical experiments show that the inorganic part of enamel and dentine is readily acted on by certain substances. There is a great variety in this process which you readily understand. Sometimes this calcareous material is dissolved out and the organic material left behind; this presents a variety of appearances. It is sometimes found that the residium is tough and leathery, and is removed by the operator in gelatinous flakes; at other times it is easily removed, and comes out in small fragments. These differences occur mainly from the character of the material that produces the decalcification. There are several materials that will dissolve this decalcareous matter. The tearing down of the teeth is an inverse operation to building them up. In building up, the organic is first formed, and next the inorganic. In decay, the first thing taken away is the inorganic, and then the organic. The bonds that hold the elements, constituents, together, are worth studying, and in studying these we learn something of the order of the process of decay.

The bonds that maintain the structural integrity of the teeth are: first, the vital, second, the chemical, and third, the mechanical; the latter depending upon the first two, as all mechanical manifestation is dependent upon chemical force

When the vital principle is removed from a tooth, the chemical force soon begins to be impaired, and disintegration from the first is a comparatively easy process. The vital force, when present, holds the peculiar chemical affinities that exist here in its grasp, and the latter is only at its best when thus dominated. I make this brief reference to this principle that we may have it in mind in the future.

Dr. Rehwinkel: It seems that Dr. Watt is not fully understood. Dr. Smith leads us to understand that he says the three acids will produce decay. Dr. Watt says such and such an acid is a nascent condition. We use strong mineral acids in and about the teeth. Suppose you take a silver teaspoon and manufacture nitric acid in it. You do it at the expense of the spoon. The very fact of the manufacture of that acid in the mouth goes to prove his theory; the teeth represent the silver spoon, and that is what he means by acid in a nascent condition.

In regard to antiseptics it is hard to make a choice. One week we hear that carbolic acid is best, but next, we find it away down in the list. Mr. Bates, of England, placed glycerine as one of the finest antiseptics. Then we have the oil of eucalyptus: the smell of this is very disagreeable, to some as unpleasant as creosote, and where it is confined in a tooth for two or three days it produces a very disagreeable taste and smell. After a while we will get something more effectual, but now everything seems to be turned around. We have a new remedy every month, and we all jump in and try it until the next thing comes. We are too much inclined to take everything for granted. Investigators are enthusiastic, but it might be as well for us to take these new remedies with a good deal of caution.

Dr. J. H. Warner: Pure beech wood creosote is far preferable to carbolic acid in all treatment of alveolar abscess. The reason is that creosote does not coagulate albumen of which the pus is largely composed, while it is coagulated by carbolic acid or any other acid, or by heat or alcohol. If carbolic acid is injected into an abscess the albumen is coagulated, the watery serum passes away through the fistulous opening, or it is absorbed by the tissues while the albumen and acid form a solid substance of cheesy consistency which is held *in situ* by the bony walls, and for a time becomes encysted, forming what is called a residual cyst. The cure for a time seems perfect but the abscess almost invariably recurs sooner or later when treated with carbolic acid. This can be avoided by using the poor beech wood creosote, something that is extremely hard to obtain, as nearly all so-called creosote is largely adulterated with carbolic acid. A twenty per cent. solution of creosote in glycerine offers as valuable a compound as any for treatment of alveolar abscess, but both the ingredients should be absolutely pure.

Dr. Taft: That thought is worth our attention. I did not refer to this variety of decay. It may be true that this variety should be treated with acid; but I would advise care in using sulphuric acid, for it might cause trouble. Dr. Smith is right when he says that anti-acids are useless; you might as well rub chalk on the teeth to arrest it. The rational treatment is a systematic change, so that lactic acid will not be produced. It does not depend on the substance of the teeth, but it comes from the condition of the mouth and of the system. There are several manifestations of this decay. Sometimes it affects only the enamel; sometimes the enamel and dentine both. Sometimes it appears as though planed and chiseled off, and sometimes grooves are formed; they are often cut down so deep that the tongue cannot come in contact with them. How are these pits made? They are not produced by bacteria. I have before spoken of a case where the teeth wasted away, so that in a few months they were destroyed in a large measure; but by change of diet and giving up milk, the process was arrested.

Dr. Rehwinkel: Dr. Bruck, of Breslaw, Germany, uses Galvano Kaustick; by touching the grooves with hot platinum wire, a crust of carbon is produced which effectually stops this wasting. I have seen many of those cases, and one of the most wonderful occurred about four years ago: It was wasting of the teeth, and it was like hanging an icicle in the sum ; the teeth literally melted away as it were. When the teeth first camc under my notice, I saw at once they would go, and in about five months I had nothing to do but cut off the crowns and put artificial crowns on the roots, and then it went on, and two of the roots had to be taken out. I have seen ridges and niches—all the phenomena of this decay—but I never saw anything so marked or so rapid in its progress as this case. She had some molars and bicuspids left, and I will be anxious to see, and hear and know, if this wasting process has gone on. I can only repeat, that the ant-acid treatment will give relief. I have found that magnesia, carb. soda, or prep. chalk will give relief, but have never yet seen anything that will stop the action. I think the cases would justify one in using anything that would form an artificial crust, because, under the best of circumstances, it is only a question of time when the teeth will go. You tell your patients to give up milk and sugar, but will they do it ?

Dr. Whitney: In examining the specimens at Peabody Museum, all of the decay would be found to be caused by chemical abrasion. I found, from the examination of many skulls, that decay was caused originally from chemical abrasions. I found it markedly so with the Chinese—with their diet of rice.

Dr. Berry: That agrees with Dr. Watts' theory, that when ammonia is formed it takes up the carbonic acid. —*Transactions of the Ohio State Dental Society*.

